# Using Two-Step Cluster Analysis and Latent Class Cluster Analysis to Classify the Cognitive Heterogeneity of Cross-Diagnostic Psychiatric Inpatients

**DOI:** 10.3389/fpsyg.2020.01085

**Published:** 2020-06-10

**Authors:** Mariagrazia Benassi, Sara Garofalo, Federica Ambrosini, Rosa Patrizia Sant’Angelo, Roberta Raggini, Giovanni De Paoli, Claudio Ravani, Sara Giovagnoli, Matteo Orsoni, Giovanni Piraccini

**Affiliations:** ^1^Department of Psychology, University of Bologna, Bologna, Italy; ^2^AUSL della Romagna, SPDC Psychiatric Emergency Unit, Cesena, Italy

**Keywords:** two-step cluster analysis, latent class cluster analysis, cognitive functioning, psychiatric inpatients, cluster analyses

## Abstract

The heterogeneity of cognitive profiles among psychiatric patients has been reported to carry significant clinical information. However, how to best characterize such cognitive heterogeneity is still a matter of debate. Despite being well suited for clinical data, cluster analysis techniques, like the Two-Step and the Latent Class, received little to no attention in the literature. The present study aimed to test the validity of the cluster solutions obtained with Two-Step and Latent Class cluster analysis on the cognitive profile of a cross-diagnostic sample of 387 psychiatric inpatients. Two-Step and Latent Class cluster analysis produced similar and reliable solutions. The overall results reported that it is possible to group all psychiatric inpatients into Low and High Cognitive Profiles, with a higher degree of cognitive heterogeneity in schizophrenia and bipolar disorder patients than in depressive disorders and personality disorder patients.

## Introduction

The traditional categorical nosology which mostly characterizes both research and clinical activity in psychology and psychiatry has been largely criticized in favor of a dimensional approach, which may better reflect the overlapping features of different disorders (Ivleva et al., 2012; Owoeye et al., 2013; van Os and Reininghaus, 2016). Cognitive impairment reflects one of the aspects shared by many psychiatric disorders, and it presents important overlaps with epidemiological, symptomatologic, and biological measures, as well as other risk factors (Smith and Weissman, 1992; Berrettini, 2000; Cosgrove and Suppes, 2013; Cross-Disorder Group of the Psychiatric Genomics Consortium, 2013; Owoeye et al., 2013; Tamminga et al., 2014; Pearlson, 2015). The heterogeneity of cognitive profiles found among psychiatric patients has been reported to carry significant information about biomarkers, etiologies, and clinical factors (Mesholam-Gately et al., 2009; Bora, 2016), and about prognosis and treatment planning (Burdick et al., 2014; Lewandowski et al., 2014), which might have important implications for their treatment and prognosis (Cochrane et al., 2012). Interestingly, these findings are in line with the so-called genetic overlap among schizophrenia, bipolar disorder, depression, and personality disorder diagnosis that has been documented so far in different studies (Witt et al., 2017; Gandal et al., 2019). However, how to best characterize such cognitive heterogeneity across or within specific diagnostic categories in an informative way is still a matter of debate, and the use of well-suited statistical techniques to achieve stable and robust conclusions on this issue appears critical.

Clustering techniques can serve this purpose by identifying homogeneous subgroups presenting similar characteristics within a large cross-diagnostic sample (Allen and Goldstein, 2013). Amongst the several approaches available, the Two-Step cluster analysis (Chiu et al., 2001; Bacher et al., 2004) and the Latent Class cluster analysis appear to be well suited for clinical data, as they can handle ordinal as well as nominal variables, which can be more informative for clinical practice (Kent et al., 2014). Indeed, data obtained from classical neuropsychological tests are not purely quantitative and are better represented as nominal measures, i.e., classifying subjective performance according to normative values that specify whether the score is “above,” “within,” or “below” the normative range. Nevertheless, the most commonly used clustering methods adopted by previous studies investigating cognitive profiles of psychiatric inpatients are either hierarchical (Goldstein and Shelly, 1987; Hermens et al., 2011; Cotrena et al., 2017; Van Rheenen et al., 2017; Crouse et al., 2018; Lewandowski et al., 2018) or k-means (Lee et al., 2017). However, such methods present several limitations, like applicability to continuous variables only, assumption of normality of distribution, and an arbitrary choice of the number of clusters (Bacher et al., 2004; Matthiesen, 2010; Everitt, 2011; Mooi and Sarstedt, 2011).

From a detailed examination of the cluster solutions proposed from previous literature (Supplementary Table S1) on major psychiatric diagnoses, most studies reported either three (Hermens et al., 2011; Lee et al., 2015; Cotrena et al., 2017; Van Rheenen et al., 2017; Crouse et al., 2018) or four clusters (Goldstein and Shelly, 1987; Lewandowski et al., 2014, 2018; Reser et al., 2015), while only a few found two clusters (Lee et al., 2017). In all these studies, executive functions seemed to be the most important measures to explain the heterogeneity of psychiatric patients’ cognitive profiles. Most studies focused on only one or two diagnostic categories, like schizophrenia and bipolar disorder (Goldstein and Shelly, 1987; Heinrichs and Zakzanis, 1998; Dawes et al., 2011; Hermens et al., 2011; Allen and Goldstein, 2013; Burdick et al., 2014; Cotrena et al., 2017; Lee et al., 2017; Roux et al., 2017; Van Rheenen et al., 2017; Crouse et al., 2018; Kollmann et al., 2019), with a few exceptions (Hermens et al., 2011; Lewandowski et al., 2014, 2018; Lee et al., 2015; Reser et al., 2015), thus limiting potential information about the differences and similarities between different diagnoses. Indeed, despite personality disorder being characterized by cognitive impairments similar to those presented by other psychiatric dysfunctions, like memory, attention, language, and executive functions (Dinn and Harris, 2000; Morgan and Lilienfeld, 2000; Dell’Osso et al., 2010; Cochrane et al., 2012; Rosell et al., 2014; Fineberg et al., 2015; Koch and Exner, 2015; McClure et al., 2016), these patients have been inexplicably neglected in this line of research.

Based on these considerations, the general goal of the present study was to identify subgroups of psychiatric inpatients based on cognitive nominal measures assessed in a large cross-diagnostic cohort (*N* = 387) including Schizophrenia Spectrum and Other Psychotic Disorders (SZ), personality disorders (PD), bipolar and related disorders (BD), and depressive disorders (DD). More specifically, we aimed to verify the best solution among those previously reported in the literature (ranging from two to four clusters; see Supplementary Table S1). The presence of a single cluster for all the diagnoses would suggest that all patients share a unique cognitive profile. The presence of two or more clusters would suggest the presence of different cognitive endophenotypes (e.g., preserved/impaired performances in specific cognitive domains or within specific diagnoses). To achieve a stable and robust solution, we provided several methodological and statistical improvements that allowed overcoming the limitations of previous similar studies (Hermens et al., 2011; Reser et al., 2015; Van Rheenen et al., 2017; Crouse et al., 2018). In particular: the stability of the clustering solution (Kraus et al., 2011) was checked by directly comparing two different techniques—Two-step and Latent Class cluster analysis—on several indexes of fit [Akaike information criterion (AIC), Bayes information criterion (BIC), and entropy]; the external validity of the solution was tested by comparing the obtained clustering solution on a different set of cognitive tests; the internal validity of the clustering solution was evaluated by running the same cluster analysis within each diagnostic subsample.

## Materials and Methods

### Participants

Three hundred and eighty-seven participants were recruited from the Psychiatric Emergency Unit of the Health Clinical Service Azienda USL della Romagna (Cesena, Italy). Following the DSM-5 and ICD-10 criteria, patients with SZ, PD, BD, and DD were included in the study. The Mini-International Neuropsychiatric Interview (Sheehan et al., 1998) and the Structured Clinical Interview (First Michael et al., 1996) were used to confirm the psychiatric diagnosis. Exclusion criteria were insufficient Italian language skills, presence of neurological disorders, and severe visual or verbal impairments.

The participants were 189 males and 198 females with a mean age of 45.7 years. All the four diagnoses included were sufficiently represented numerically: 28% (*n* = 110) of the subjects had a diagnosis of SZ, 35% (*n* = 134) had a diagnosis of BD, 24% (*n* = 93) had a diagnosis of DD, and 13% (*n* = 50) had a diagnosis of PD. The demographic and clinical characteristics of the whole sample are reported in Table 1. Differences in cognitive performance among diagnoses are reported in the Supplementary Information and Supplementary Figure S1.

**TABLE 1:** Demographic and clinical characteristics of the whole sample.

		**Participants *n* = *387***
***Age*** *mean (S.D.; range)*		45.7 (14.1; 17–80)
***Gender*** *n* (%) M/F (% M)		189/198 (48.8)
***Nationality*** *n* (%) Italian/others (% Italian)		292/95 (76.2)
***Education*** *n* (%)	Primary school	17 (4.3)
	Secondary school	114 (29.4)
	High school	116 (30.0)
	Degree	23 (6.0)
	Missing	117 (30.3)
***Diagnosis*** *n* (%)	Schizophrenia Spectrum and Other Psychotic Disorders	110 (28)
Bipolar and Related Disorders		134 (35)
Depressive Disorders		93 (24)
Personality Disorders		50 (13)
***BPRSa*** *mean (S.D.)*		48.2 (10.3)
***BPRSd*** *mean (S.D.)*		35.2 (7.5)
***HoNOS*** *mean (S.D.)*		30.4 (6.5)

*HoNOS, Health of the Nation Outcome Scales; BPRSa, Brief Psychiatric Rating Scale administered at admission; BPRSd, Brief Psychiatric Rating Scale administered at discharge.*

All procedures complied with the ethical standards of the relevant national and institutional committees on human experimentation and with the Helsinki Declaration of 1975, as revised in 2008. The study was approved by the Research Ethical Committee of the AUSL Romagna (Regional Health Clinical Service). Written informed consent was acquired from each participant or, whenever necessary, from a parent or legal guardian.

Information about medication at the time of assessment was obtained from the medication list. All the patients were taking various combinations of mood stabilizers, antipsychotics, and antidepressants.

### Cognitive and Clinical Assessment

The inpatients, admitted during the acute phase of illness, were recruited during the hospitalization. A team of psychologists and psychiatrists performed cognitive and clinical assessments. The complete assessment lasted approximately 3 h (see Supplementary Information for a comprehensive description of the tests used in the study).

The severity of symptomatology was measured at admission and at discharge with the Brief Psychiatric Rating Scale Expanded Version 4.0 (BPRS) (Ventura et al., 1993), while health and social functioning were measured with the Health of the Nation Outcome Scales—Roma (HoNOS) (Morosini et al., 2003).

Each patient completed two self-report questionnaires concerning the quality of life and the level of disability experienced during their daily life, respectively, the *World Health Organization Quality of Life—BREF* (WhoQoL) (Skevington et al., 2004) and the *World Health Organization Disability Assessment Schedule 2.0—36 items* (WhoDAS) (Üstün, 2010). The *UKU Side Effect rating scale* (Lingjaerde et al., 1987) was administered to evaluate the severity of pharmacological treatment side effects.

The *Tower of London—Drexel University* (ToL) (Culbertson and Zillmer, 2001) was used to assess planning abilities and problem-solving. The *Modified Wisconsin Card Sorting Test* (MCST) (Caffarra et al., 2004) was used to analyze the tendency toward perseveration and shifting. The *Attentional Matrices* (AM) (Spinnler and Tognoni, 1987) test was applied to evaluate selective visual attention. The *Stroop Word Interference Test* (STROOP) (Caffarra et al., 2002) was used as an index of selective attention, inhibitory control, and processing speed. The Italian standardized version of *Raven’s Colored Progressive Matrices* (CPM-47) (Pruneti et al., 1996) was used to evaluate fluid intelligence.

A set of other cognitive measures was collected to explore the external validity of the clusters. Global cognitive functioning was assessed using the Mini Mental State Examination (MMSE) (Folstein et al., 1975) and the Clock Drawing Test (CDT) (Watson et al., 1993). Mental flexibility and verbal intelligence were assessed using Test dei Giudizi verbali e dei Compiti Astratti (Verbal abilities and abstract thinking test, GCA) (Spinnler and Tognoni, 1987). The Digit Span (Orsini et al., 1987) was used to assess short-term memory (SPAN Forward) and working memory (SPAN Backward).

For each test included in the cognitive assessment, detailed information about the purpose of the instrument, number of items and subscales, response recording method, administration time, scores, and psychometric properties is reported in the Supplementary Information.

### Statistical Analysis

The variables used in the present study were standardized according to the normative scores available for each test (see Supplementary Information) by applying the following formula: *z* = (*x* - μ)/σ, where *x* is the subject’s raw score, μ represents the average obtained in the normative population, and σ is the normative population standard deviation. Then, following the indication of common clinical practice and the general guidelines for neuropsychological assessment (Mitrushina et al., 2005), the standardized scores were transformed into three categories: scores below the 10th percentile (corresponding to z score < -1.3) indicated cognitive deficit; scores equal or above the 10th and below the 90th percentile (corresponding to z score > = -1.3 and < 1.3) indicated normal cognitive functioning; and scores equal to or above the 90th percentile (corresponding to z score > = 1.3) indicated superior cognitive ability.

The variables included in both cluster analyses were: ToL (Total Number of Moves, Number of Correct Moves, Rule Violations, and Time Violations subscales), MCST (number of categories and Perseverative Errors subscales), CPM-47 total score, AM total score, and STROOP (Time and Errors subscales). The *Two-Step cluster analysis* is a hybrid approach which first uses a distance measure to separate groups and then a probabilistic approach (similar to latent class analysis) to choose the optimal subgroup model (Gelbard et al., 2007; Kent et al., 2014). Such a technique presents several advantages compared to more traditional techniques, like determining the number of clusters based on a statistical measure of fit (AIC or BIC) rather than on an arbitrary choice, using categorical and continuous variables simultaneously, analyzing atypical values (i.e., outliers), and being able to handle large datasets (Chiu et al., 2001; Bacher et al., 2004; Gelbard et al., 2007; Mooi and Sarstedt, 2011; Kent et al., 2014). Comparative studies regarded Two-Step cluster analysis as one of the most reliable in terms of the number of subgroups detected, classification probability of individuals to subgroups, and reproducibility of findings on clinical and other types of data (Bacher et al., 2004; Gelbard et al., 2007; Kent et al., 2014). The Two-Step cluster analysis was implemented in IBM SPSS Statistics (version 23.0) (Chiu et al., 2001; Bacher et al., 2004). In the first step (pre-clustering), a sequential approach is used to pre-cluster the cases based on the definition of dense regions in the analyzed attribute-space. In the second step (clustering), the pre-clusters are statistically merged in a stepwise way until all clusters are in one cluster.

The *Latent Class cluster analysis* consists of finding latent factors or class referred to a specific model that, from manifest variables, determines the differences among groups of subjects (Vermunt and Magidson, 2002, 2009; Allen and Goldstein, 2013; Kent et al., 2014). This approach is a model-based clustering technique in which, starting from the distribution of the data, each case or observation is probabilistically clustered into a latent class (McLachlan and Peel, 2000; Vermunt and Magidson, 2009). The model parameters are estimated as the proportion of observations in each latent class, and they are determined by the conditional probability of observing each response for each manifest variable in a given class. The cases presenting similar responses to the manifest variables are more likely included within the same latent class. Importantly, this approach is suitable for fitting ordinal manifest variables as well as nominal. The Latent Class cluster analysis was implemented using the R package “poLCA” (Haughton et al., 2009; Linzer and Lewis, 2011; Flynt and Dean, 2016). This procedure aims to fit a model in which any confounding between the manifest variables can be explained by a single unobserved “latent” categorical variable. Local independence is assumed to estimate a mixture model of latent multi-way tables.

Following a parsimony criterion, the best clustering solution was considered the one with the best balance between the number of clusters considered and the corresponding fit. Based on previous literature (see Supplementary Table S1), solutions ranging from two to four clusters were considered. BIC, AIC, and entropy were first calculated for each cluster solution and then used to find the greatest change in distance between two cluster solutions. BIC, AIC, and entropy change were calculated as the difference between two cluster solutions starting from the most parsimonious (one cluster) to the less parsimonious (four clusters), thus obtaining three values (2vs1, 3vs2, and 4vs3). The best cluster solution was considered the one with the strongest change and the lower number of clusters. This allowed evaluating the most parsimonious cluster solution presenting the best fit. Such a procedure was performed automatically for the Two-Step cluster analysis and implemented via a custom-made script implemented in R for the Latent Class cluster analysis.

Aiming for a detailed description of the selected clustering solution, the clusters were compared based on clinical and psychosocial functioning using a general linear model on the following continuous variables: severity of psychiatric symptoms (HoNOS and BPRS), side effects of pharmacological treatment (UKU), duration of hospitalization, number of hospitalizations, and quality of life (WhoQoL and WhoDAS). A chi-squared test was used to compare the frequency of diagnosis between the two clusters.

The external validity of the clustering solutions was verified by comparing the clusters (independent variable) on a different set of cognitive tests (dependent variables), including global cognitive functioning (MMSE and CDT), mental flexibility and verbal intelligence (GCA), short-term memory (Digit Span Forward), and working memory (Digit Span Backward). General linear models were used for normally distributed variables (MMSE and CDT). Mann–Whitney tests were used for non-normally distributed variables (GCA and Digit Span Forward and Backward).

The internal validity of the clustering solution was evaluated by dividing the sample according to the diagnosis and running both the Two-Step and Latent Class cluster analysis on each subsample. Cohen’s Kappa statistic was calculated to test the degree of agreement between the cluster assignment for each subject when considered in the cross-diagnostic sample and within the single diagnostic subsample.

## Results

**FIGURE 1:**
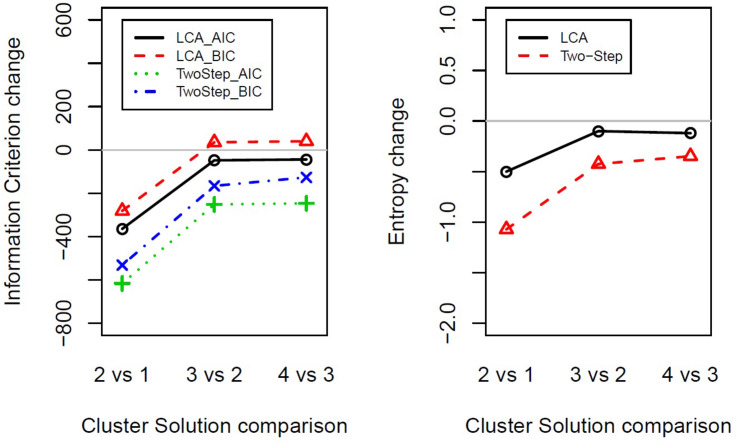
Indexes of fit changes obtained from Latent Class cluster analysis and Two-Step cluster analysis for solutions ranging from one to four clusters. The panels show the change in information criterion (left) or entropy (right) between two close clusters’ solutions (e.g., 2vs1 shows two-cluster solution minus one-cluster solution). LCA, Latent Class cluster analysis; TwoStep, Two-Step cluster analysis; BIC, Bayesian information criterion; AIC, Akaike information criterion.

The results that emerged from both the Two-Step and the Latent Class cluster analysis reported a two-cluster classification as the optimal solution for the data considered in the present study. That is, following a parsimony criterion (see the *Statistical Analysis* section), the two-cluster solution presented the greatest BIC, AIC, and entropy change between the two closest clusters at each stage (Figure 1 and Supplementary Table S2). Following the principle of parsimony, the best cluster solution is the one with the highest value of the difference between two indexes of n cluster and n plus one cluster. This way to select the best cluster solution allows evaluating the improvement of homogeneity within each cluster and the heterogeneity between the clusters from one cluster to n cluster by adding one cluster at each step.

**FIGURE 2:**
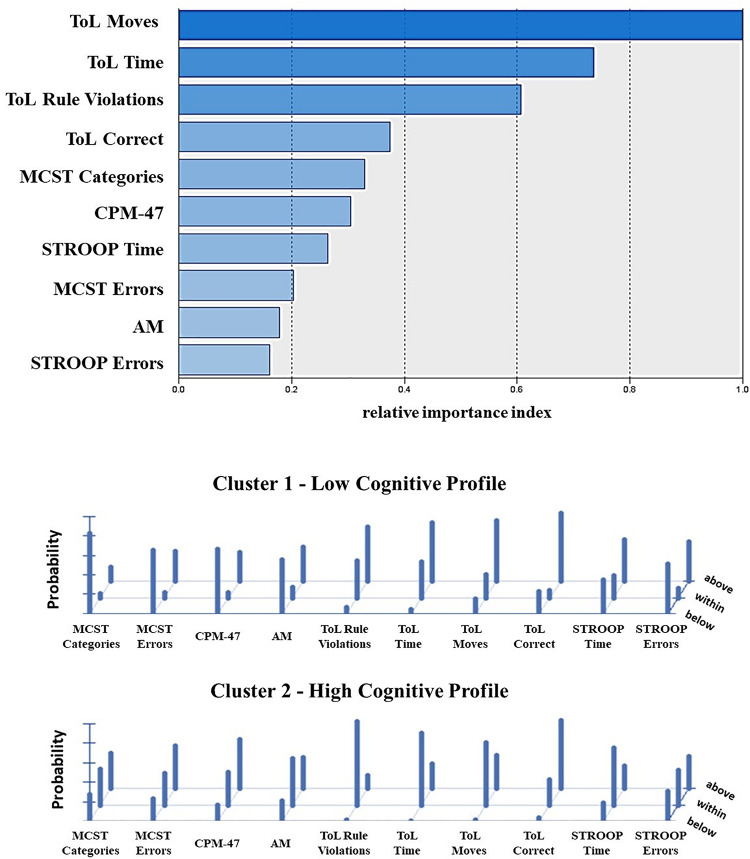
Contribution of the single cognitive tests to the clustering solution as reported from the Two-Step **(top)** and Latent Class cluster analysis **(bottom)**. The top panel shows the index of relative importance of each cognitive test as identified by the Two-Step cluster analysis. The panel on the bottom shows the conditional item response probabilities for the two clusters identified by the Latent Class cluster analysis. Performance class of score below (z score < –1.3) average (z score between –1.3 and +1.3) and above (z score > 1.3) the normative sample. MCST, Modified Wisconsin Card Sorting Test; CPM-47, Raven’s Colored Progressive Matrix; AM, attentional matrices; ToL, tower of London—Drexel University test; STROOP, Stroop Word Interference Test.

The frequency distribution of performances scoring below, within, and above the normative sample for each cognitive test was examined to define the composition of the two clusters (Table 2). The results showed a significantly higher presence of performances classified as “below” in one cluster and “within” or “above” in the other cluster, for both the Two-Step and the Latent Class clustering solutions (Table 2). Consequently, one group was defined as the Low Cognitive Profile cluster (including 48% of subjects for the Two-Step clustering solution and 52% of subjects for the Latent Class clustering solution), and the other group was defined as the High Cognitive Profile cluster. The contribution of each cognitive test to such a clustering solution is represented in Figure 2. For the Latent Class cluster analysis, the major cognitive differences between clusters concerned perseveration and shifting abilities (MCST), fluid intelligence (CPM-47), and selective visual attention (AM), while for the Two-Step cluster analysis, the major cognitive differences between clusters concerned planning abilities and problem-solving (ToL). Since the two clusters reported differences in age (*F*_2,304_ = 0.63; *p* = 0.533; partial η^2^ = 0.004) and education (*F*_2,304_ = 2.64; *p* = 0.073; partial η^2^ = 0.017), these two variables were introduced as covariates in all analyses. A general linear model was applied to verify whether the clusters differed in clinical and psychosocial functioning. Although with some discrepancies between the Two-Step and the Latent Class clustering solutions, the Low Cognitive Profile cluster generally reported higher severity of symptoms (HoNOS and BPRS at admission and discharge), higher side effects of pharmacological treatment (UKU), lower improvement in BPRS symptom severity between admission and discharge, and longer duration of hospitalization than the High Cognitive Profile cluster (Table 3). No differences were found on measures of quality of life (WhoQoL and WhoDAS) and the number of hospitalizations (Table 3). The diagnoses were differently represented in the two clusters. Most of the schizophrenia and bipolar disorder patients were similarly distributed between the High and Low Cognitive Profile clusters, while most depressive disorder and personality disorder patients were more represented in the High Cognitive Profile cluster (Table 3).

**TABLE 2:** Description of the two clusters according to the number and percentage of cases scoring below, within, and above the normative scores for each cognitive test.

		**Cluster 1**			**Cluster 2**			
		***Low Cognitive Profile***			***High Cognitive Profile***			
	***Tests N (%)***	**Below**	**Within**	**Above**	**Below**	**Within**	**Above**	**χ^2^**
Two-Step	MCST categories	58(29)	69(34)	76(37)	17(9)	26(14)	141(77)	60.56; *p* < 0.001
	MCST errors	55(27)	82(40)	66(33)	14(8)	61(33)	109(59)	37.17; *p* < 0.001
	CPM-47	58(29)	91(45)	54(27)	12(7)	60(33)	112(61)	56.06; *p* < 0.001
	AM	81(40)	64(32)	58(28)	28(15)	64(35)	92(50)	32.62; *p* < 0.001
	ToL Rule Violations	173(85)	27(13)	3(1)	60(33)	112(61)	12(7)	111.52; *p* < 0.001
	ToL N of correct moves	52(26)	147(72)	4(2)	10(5)	124(67)	50(27)	68.82; *p* < 0.001
	ToL Time Violations	167(82)	35(17)	1(1)	43(23)	133(72)	8(4)	135.22; *p* < 0.001
	ToL total N of moves	150(74)	52(26)	1(0)	13(7)	138(75)	33(18)	183.7; *p* < 0.001
	STROOP Time	112(55)	55(27)	36(18)	39(21)	75(41)	70(38)	48.46; *p* < 0.001
	STROOP Errors	64(32)	75(37)	64(32)	20(11)	67(36)	97(53)	29.4; *p* < 0.001
Latent Class	MCST categories	69(38)	67(36)	48(26)	6(3)	28(14)	169(83)	135.8; *p* < 0.001
	MCST errors	60(33)	82(45)	42(22)	9(4)	61(30)	133(66)	87.38; *p* < 0.001
	CPM-47	63(34)	92(50)	29(16)	7(3)	59(29)	137(68)	121.64; *p* < 0.001
	AM	91(49)	56(31)	37(20)	18(9)	72(35)	113(56)	88.68; *p* < 0.001
	ToL Rule Violations	157(85)	25(14)	2(1)	76(37)	114(56)	13(7)	92.50; *p* < 0.001
	ToL N of correct moves	47(26)	131(71)	6(4)	15(7)	140(69)	48(24)	48.67; *p* < 0.001
	ToL Time Violations	138(75)	45(24)	1(1)	72(35)	123(61)	8(4)	61.62; p < 0.001
	ToL total N of moves	118(64)	62(34)	4(2)	45(22)	128(63)	30(15)	74.75; *p* < 0.001
	STROOP Time	109(59)	40(22)	35(19)	42(21)	90(44)	71(35)	60.40; *p* < 0.001
	STROOP Errors	66(36)	58(32)	60(33)	18(9)	84(41)	101(50)	41.80; *p* < 0.001

*Performance class of score below (z score < −1.3) average (z score between −1.3 and +1.3) and above (z score > 1.3). MCST, Modified Wisconsin Card Sorting Test; CPM-47, Raven’s Colored Progressive Matrix; AM, Attentional Matrices; ToL, Tower of London—Drexel University test; STROOP, Stroop Word Interference Test.*

**TABLE 3:** Clinical characteristics and distribution of diagnoses in the two clusters.

			**Cluster 1**	**Cluster 2**		
			***Low cognitive profile***	***High cognitive profile***	**Statistic**	
Two-Step	Test *mean (s.e.)*	HoNOS	31.87(0.51)	29.28(0.56)	*F*_1,321_ = 11.86	*p* = 0.001
		BPRSa	49.67(0.74)	46.55(0.78)	*F*_1,321_ = 8.26	*p* = 0.004
		BPRSd	36.44(0.57)	34.31(0.63)	*F*_1,321_ = 6.23	*p* = 0.013
		BPRSa-d	12.32(0.52)	12.78(0.57)	*F*_1,313_ = 8.81	*p* = 0.003
		UKU	3.30(0.19)	3.01(0.19)	*F*_1,175_ = 1.2	*p* = 0.276
		*WhoQoL*	81.68(1.44)	78.5(1.54)	*F*_1,338_ = 2.22	*p* = 0.137
		*WhoDAS*	80.72(2.27)	83.45(2.68)	*F*_1,261_ = 0.60	*p* = 0.438
	Hosp.	Duration	13.98	12.27	*F*_1,385_ = 3.99	*p* = 0.05
		Number	1.68	1.82	*F*_1,385_ = 0.83	*p* = 0.36
	Diagnosis *N (%)*	*BD*	77(57)	57(43)	$\chi_{3}^{2}\text{=}16.58$	*p* = 0.001
		*DD*	41(44)	52(56)		
		*PD*	18(36)	32(64)		
		*SZ*	67(61)	43(39)		
Latent Class	Test *mean (s.e.)*	HoNOS	32.72(0.52)	28.81(0.042)	*F*_1,321_ = 28.56	*p* < 0.001
		BPRSa	50.20(0.74)	46.45(0.79)	*F*_1,321_ = 11.53	*p* = 0.001
		BPRSd	37.13(0.06)	33.91(0.49)	*F*_1,321_ = 14.75	*p* < 0.001
		BPRSa-d	13.50(0.53)	11.80(0.56)	*F*_1,321_ = 1.81	*p* = 0.17
		UKU	3.51(0.14)	2.88(0.12)	*F*_1,175_ = 5.74	*p* = 0.018
		*WhoQoL*	80.85(1.34)	79.64(1.44)	*F*_1,338_ = 0.33	*p* = 0.568
		*WhoDAS*	79.64(0.10)	84.03(2)	*F*_1,261_ = 1.61	*p* = 0.21
	Hosp.	Duration	14.4	12	*F*_1,385_ = 7.56	*p* = 0.006
		Number	1.8	1.7	*F*_1,385_ = 0.48	*p* = 0.49
	Diagnosis *N (%)*	*BD*	73(54)	61(46)	$\chi_{3}^{2}\text{=}30$	*p* < 0.001
		*DD*	26(28)	67(72)		
		*PD*	19(38)	31(62)		
		*SZ*	66(60)	44(40)		

*Hosp., hospitalization; BPRSa-d, Brief Psychiatric Rating Scale difference between BPRSa and BPRSd; UKU, UKU side effect rating scale; WhoQoL, world health organization quality of life—BREF scale; Who DAS, world health organization disability assessment schedule; SZ, schizophrenia spectrum and other psychotic disorders; BD, bipolar and related disorders; DD, depressive disorders; PD, personality disorders.*

The analysis for the external validity confirmed the presence of poorer global functioning, short-term memory, working memory, and mental flexibility and verbal intelligence in the Low Cognitive Profile cluster as compared to the High Cognitive Profile cluster. Such differences were present in both the clustering solutions identified by means of Two-Step cluster analysis (MMSE, *F*_1,297_ = 60.72, *p* < 0.001, partial η^2^ = 0.170; CDT, *F*_1,123_ = 19.21, *p* < 0.001, partial η^2^ = 0.135; GCA, *U* = 6,314.00, *p* < 0.001; SPAN Forward, *U* = 8,130.50, *p* = 0.018; SPAN Backward, *U* = 7,181.50, *p* < 0.001) and Latent Class cluster analysis (MMSE, *F*_1,296_ = 65.83, *p* < 0.001, partial η^2^ = 0.18; CDT, *F*_1,122_ = 24.67, *p* < 0.0001, partial η^2^ = 0.17; GCA, *U* = 6,314.00, *p* < 0.001; SPAN Forward, *U* = 8,000, *p* < 0.001; SPAN Backward, *U* = 7,000, *p* < 0.001). The Low Cognitive Profile performed worse than the High Cognitive Profile in all the tests: MMSE, Low Cognitive Profile = 26.16 (S.E. = 0.21) vs High Cognitive Profile 28.36 (SE.19); CDT, Low Cognitive Profile = 10.27 (S.E. = 0.39) vs High Cognitive Profile 12.70 (SE.39); GCA, Low Cognitive Profile mean rank = 114.47 vs High Cognitive Profile mean rank = 189.12; SPAN-Forward, Low Cognitive Profile mean rank = 128.04 vs High Cognitive mean rank = 160.50; SPAN Forward mean rank = 120.74 vs high cognitive mean rank = 166.39. These results showed that in all the tests, the Low Cognitive Profile obtained with Two-Step cluster analysis performed worse than the High Cognitive Profile.

**FIGURE 3:**
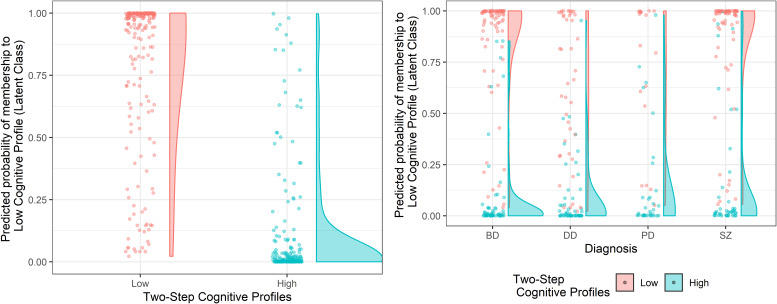
Cluster assignment according to the Two-Step clustering solution as a function of the predicted probability of cluster membership of the Latent Class clustering solution, on the cross-diagnostic sample and within each diagnosis. The left panel represents the clustering solutions obtained on the cross-diagnostic sample. The panel on the right represents the clustering solutions obtained within each diagnosis. SZ, Schizophrenia Spectrum and Other Psychotic Disorders; BD, Bipolar and Related Disorders; DD, Depressive Disorders; PD, Personality Disorders.

The internal validity of the clustering solution was verified by applying the same cluster procedures on each of the four diagnostic groups separately. The results reported the two-cluster classification as the optimal solution within each diagnosis (Supplementary Figure S2 and Supplementary Table S2), thus confirming the result obtained on the cross-diagnostic sample as stable and consistent. Cohen’s Kappa statistics showed a significant agreement between the results of the whole cross-diagnostic sample and those emerging from the single diagnostic subsamples for both the Two-Step (Kappa = 0.66; *p* < 0.001) and the Latent Class (Kappa = 0.72; *p* < 0.001) cluster analysis. Patients were re-classified according to the cross-diagnostic solution in 83% of cases for the Two-Step clustering solution and in the 87% of cases for the Latent Class clustering solution. Overall, the two clusters obtained within each diagnosis were confirmed as being characterized by a lower and a higher cognitive profile (Supplementary Tables S3, S4). However, important differences were observed between the diagnoses. Indeed, for both clustering techniques, while schizophrenia and bipolar disorder patients showed a clear-cut separation and a fairly even distribution of subjects between the two clusters, depressive disorder and personality disorder patients were more represented in the High Cognitive Profile cluster (Figure 3; see also Table 3), thus showing lower cognitive heterogeneity.

To support of the validation of the two cluster solutions obtained with categorical variables, we applied the Two-Step cluster analysis to quantitative data (i.e., standardized scores). Results showed that the two cluster solutions remained the best option according to AIC and BIC changes (see Supplementary Table S5).

## Discussion

The main findings here reported responded to our general aim to find reliable and robust cognitive clusters of psychiatric inpatients by comparing Two-Step and Latent Class cluster analysis. To our knowledge, despite the wide use of different cluster analyses in former literature, no study compared different clustering approaches that can handle nominal data on a cross-diagnostic sample of psychiatric inpatients. The two cluster analyses converged on finding the presence of two separate clusters (Low and High) as the most efficient and robust description of the whole sample’s cognitive profile. Importantly, clustering was not dependent on pharmacological treatment side effects, as the two clusters reported comparable levels of iatrogenic effects. Measures of internal and external validity also confirmed the two-cluster classification as the best solution.

The analysis performed within each diagnostic sample showed that while schizophrenia and bipolar disorder were similarly represented in the two clusters, depressive disorder and personality disorder patients were overrepresented in the High Cognitive Profile cluster (Figure 3 and Table 3), thus indicating a higher cognitive heterogeneity in the first two diagnostic categories than in the last two. Crucially, given the known link with biomarkers, etiologies, and clinical factors reported in the literature about cognitive heterogeneity (Burdick et al., 2014; Lewandowski et al., 2014), such differentiation can be informative for clinical practice in terms of both prognosis and treatment planning (Cochrane et al., 2012; Burdick et al., 2014; Lewandowski et al., 2014). Indeed, the two clusters resulted as different in terms of severity and improvement of the symptomatology, side effects of pharmacological treatment, and duration of hospitalization.

The number of clusters here obtained is dissimilar to most of the previous studies using cross-diagnostic samples. A direct comparison between different cluster analytic studies is always problematic, as the clustering solutions are highly sensitive to the input data and the algorithm chosen (Marquand et al., 2016). For example, due to the marked variability of neuropsychological measures used by the previous studies above mentioned, any consideration would be limited by the absence of cluster analytic studies based on the same input data but extended to different cohorts. Nevertheless, we will try to examine the main differences and similarities with previous studies, in the attempt to obtain a more general overview of the currently available evidence (Supplementary Table S1). A recent study from Lee et al. (2017) in schizophrenia and bipolar disorder patients reported two clusters (for a complete overview, see Supplementary Table S1). Conversely, most studies reported either three (Hermens et al., 2011; Lee et al., 2015; Cotrena et al., 2017; Van Rheenen et al., 2017; Crouse et al., 2018) or four clusters (Goldstein and Shelly, 1987; Lewandowski et al., 2014, 2018; Reser et al., 2015). The main reason for obtaining more than two clusters could be attributed to the inclusion of healthy subjects within the cluster analysis and the presence of verbal reasoning tests, which we excluded in favor of a deeper evaluation of executive functions, as classically reported as the most important measures to explain the heterogeneity of cognitive profiles (Goldstein and Shelly, 1987; Hermens et al., 2011; Lewandowski et al., 2014, 2018; Lee et al., 2015, 2017; Reser et al., 2015; Cotrena et al., 2017; Van Rheenen et al., 2017; Crouse et al., 2018). Relatedly, some authors indicated that intermediary clusters could reflect a degree of normal variability across measures of cognitive functioning (Binder et al., 2009) that may underpin different brain abnormalities as far as nature and severity are concerned (Demjaha et al., 2012; Woodward, 2016). However, whether the clusters characterized by selective cognitive impairment represent distinct profiles or only reflect artificial divisions along a continuum of severity is a matter of debate (Wykes and Reeder, 2005). Indeed, the results reported may, at least in part, be confounded by the statistical and methodological limitations of these studies. Indeed, in contrast with previous literature, the robustness of the selected cluster solution was here tested by comparing two clustering techniques, namely Two-Step and Latent Class cluster analysis, that can both handle nominal data and continuous data and are based on optimal BIC and AIC indexes of fit (Chiu et al., 2001; Haughton et al., 2009). These two critical points are the main strengths of the two approaches. Moreover, some specific features of each technique should be mentioned. While the Two-Step cluster analysis is based on a fixed model procedure, in the Latent Class, a probability-based classification is computed for each subject according to the specific model selected by the researcher. Therefore, in the Latent Class cluster analysis, it is possible to obtain the subjective probability membership to each cluster (Figure 3). These aspects already have been discussed in previous literature (Chiu et al., 2001), but no previous study attempted to use them as a validation method for determining the stability of the selected cluster solution. Furthermore, given the known limitations of the cluster analysis, internal and external validation of a clustering solution, as reported in the present study, is always crucial (Marquand et al., 2016). A review by Marquand et al. (2016) has well explained that applying a cluster analysis necessarily entails some heuristics, concerning the choice of algorithm, distance function, and model order, which influence the clustering solution and complicate potential quantitative comparisons between different studies and cohorts. Unfortunately, only a few cross-diagnostic studies provided a validation of the clustering solution obtained (Hermens et al., 2011; Lee et al., 2015; Reser et al., 2015; Van Rheenen et al., 2017; Crouse et al., 2018). The two clusters identified in the present study can be considered as robust since both the external and internal validity of the clustering solution were verified. That is, the Low and High Cognitive Profiles were distinguishable also when compared based on a set of cognitive measures not considered during the cluster analysis and when applying the same cluster procedure on each of the four diagnostic groups separately.

Some limitations of the present study should also be mentioned. Personality Disorder patients are slightly underrepresented in the whole sample. This limitation may have biased the results; therefore, additional studies are needed to better understand if it is possible to find specific cognitive profiles in Personality Disorder patients. Although we attempted to analyze the contribution of pharmacological treatment in the clustering solution, we could only evaluate the iatrogenic effect. Further studies are required to investigate the effect of pharmacological treatment in grouping the cognitive performance of psychiatric patients.

## Conclusion

Despite the large variety of solutions proposed by previous literature, the application and comparison of Two-Step and Latent Class cluster analysis on four possible clustering solutions (one to four clusters) allowed confirmation of the robustness of two clusters as the best representation of the cognitive heterogeneity characterizing large cross-diagnostic psychiatric inpatients. The presence of similar solutions obtained with two separate procedures suggests a combined use for future applications to maximize the criteria selection efficiency. These results have also important clinical implications. By clarifying that two subgroups of patients with low or high cognitive abilities can be identified in all the diagnostic groups, we envision the possibility to find specific phenotypes connected to executive functions. These two groups, irrespectively from the diagnosis, present different symptom severity and prognosis (better outcome and lower duration of hospitalization for those patients who are not cognitively impaired as compared to the ones with cognitive deficits). This result informs clinical practice about the fact that specific cognitive training could be proposed to psychiatric patients with low cognitive profile, and suggests that a specific cognitive evaluation could enhance the clinical effectiveness for personalized intervention.

## Data Availability Statement

The data supporting the findings of the present study can be found in the Supplementary Material.

## Ethics Statement

The studies involving human participants were reviewed and approved by Ausl della Romagna, Ethical Committee. The participants provided their written informed consent to participate in this study.

## Author Contributions

MB, SGi, CR, and GP developed the main hypothesis, and all authors contributed to the study design. Patient screening, testing, and the data collection were carried on by FA, RS, RR, GD, and GP. Data analysis was performed by SGa, FA, and MO under the supervision of MB, GP, and SGi. The main text was written by MB, SGa, and FA. All authors approved the final version.

## Conflict of Interest

The authors declare that the research was conducted in the absence of any commercial or financial relationships that could be construed as a potential conflict of interest.
