# Supplementary material for: Using Two-Step Cluster Analysis and Latent Class Cluster Analysis to Classify the Cognitive Heterogeneity of Cross-Diagnostic Psychiatric Inpatients
Source: Front Psychol. 2020 Jun 10;11:1085. doi: 10.3389/fpsyg.2020.01085 (PMC7299079; doi:10.3389/fpsyg.2020.01085)
Supplement: Supplementary file 2 [file Data_Sheet_2.PDF]

SUPPLEMENTARY MATERIALS

Description of clinical and cognitive measures

The complete list of clinical and cognitive measures is the following:

1. Brief Psychiatric Rating Scale Expanded Version 4.0 (BPRS) (Ventura et al., 1993)
2. Health of the Nation Outcome Scales – Roma (HoNOS) (Morosini et al., 2003).
3. *World Health Organization Quality of Life – BREF* (WHOQoL-BREF) (De Girolamo et al., 2000)
4. *World Health Organization Disability Assessment Schedule 2.0 – 36 items* (WHODAS) (Federici et al., 2009).
5. The *UKU Side Effect rating scale* (Lingjaerde et al., 1987).
6. Tower of London-Drexel University (ToL-DX) (Culbertson and Zillmer, 2001)
7. *Modified Wisconsin Card Sorting Test* (MCST) (Caffarra et al., 2004)
8. *Attentional Matrices* (AM) (Della Sala et al., 1992)
9. *Stroop Colour Word Interference Test* (STROOP) (Caffarra et al., 2002)
10. *Raven's Coloured Progressive Matrices* (CPM-47) (Caltagirone et al., 1995)
11. *Judgment and Verbal Abstract Tasks test* (Test dei Giudizi Verbali e dei Compiti Astratti - GCA, Spinnler and Tognoni, 1987).
12. Digit Span (SPAN) (Monaco et al., 2013)
13. Mini Mental State Examination (MMS-E) (Measso et al., 1993)
14. Clock Drawing Test (CDT) (Caffarra et al., 2011).

Brief Psychiatric Rating Scale Expanded Version 4.0 (BPRS; Ventura et al., 1993)

The severity of symptomatology was measured with the Italian version of the Brief Psychiatric Rating Scale Expanded Version 4.0 (BPRS) (Ventura et al., 1993, Italian translation and adaptation by Morosini and Casacchia, 1995). BPRS is a semi-structured interview aimed to evaluate the most common psychopathologic symptoms, with particular attention to affective, anxious and psychotic domains and allows a rapid and effective assessment of symptomatology changes in psychiatric patients. BPRS is composed of 24-items focused on common symptoms for psychotic patients and subjects with severe affective disorders with or without psychotic features. Each item can be rated on a seven-point Likert scale from 1 (Not present) to 7 (Extremely severe) by the examiner during the interview. The total score is obtained summing up the score of all items and the possible scores vary from 24 to 168 with lower scores indicating less severe psychopathology. Items from 1 to 14 require a scoring based on what is referred by the patient during the interview.

Administration time: about 20 minutes.

Psychometric properties: The Italian version of BPRS 4.0 shows a satisfactory level of inter-rater reliability ranging from .64 to .81 (Roncone et al., 1999). We measured the test retest reliability in the present sample by reporting the correlation between BPRS at admission and BPRS discharge. The results showed low reliability ($r = .48$).

Health of the Nation Outcome Scales – Roma (HoNOS) (Morosini et al., 2003).

The health and social functioning was measured with the *Health of the Nation Outcome Scales – Roma* (HoNOS) (Morosini et al., 2003). The HoNOS is a rating scale useful for the assessment of mental health and social functioning. The examiner evaluates the patient on 18 items rated on a five-point Likert scale

(1 = No problem to 5 = severe to very severe problem). The final evaluation can be expressed both with the sum score of the scores of the 18 items (ranging from 18 to 90) and with partial scores of 4 subscales. The total score represents the variable of interest in this study.

Administration time: 10 - 20 minutes.

Psychometric properties: the Italian version of HONOS showed good inter-rater reliability (weighted kappa > .71 for all items) and a good internal consistency (Cronbach's alpha =.70). Factor analysis was consistent with the division of HoNOS-Rome into four sensible factors accounting for 52% of the total variance (Morosini et al, 2003). The HoNOS criterion validity was consistent, however, the discriminant ability of the scale is modest (in discriminant function analysis, the classification procedure correctly classified 55.7% of the patients) (Gigantesco et al., 2004). We measured the test-retest reliability in the present sample by reporting the correlation between Honos at admission and Honos at discharge. The results showed high reliability (r = .72).

World Health Organization Quality of Life – BREF (WHOQoL-BREF) (De Girolamo et al., 2000)

WHOQoL-BREF (Skevington et al., 2004) is a self-report questionnaire and consists of 26 items about 4 domains: physical health (7 items), psychological (6 items), social relationships (3 items), and environment (8 items). Each item is rated on a 5-point scale from 1 (Not at all) to 5 (Completely). Higher scores denote a higher quality of life. The total score was obtained by calculating the average of the transformed score of all sections and was used as a variable of interest in this study.

Administration time: 10 - 15 minutes.

Psychometric properties: WHOQOL-BREF was developed simultaneously in 15 international centers, and the Italian version of the instrument proved to have satisfactory psychometric properties (De Girolamo et al., 2000): the WHOQOL-BREF domains has shown good internal consistency (Cronbach’s Alpha ranging from .65 to .80) and the test-retest reliability values were also good, ranging from .76 to .93. Concurrent validity between the WHOQOL-Brief and the MOS-SF-36 (Ware and Sherbourne, 1992) was satisfactory. We measured the divergent validity in the present sample correlating WHOQoL and WHODAS scores, reporting a fair high and negative correlation as expected (r = -.64).

World Health Organization Disability Assessment Schedule 2.0 – 36 items (WHODAS) (Federici et al. 2009).

WHODAS 2.0 (Federici et al., 2009) is a self-report questionnaire measuring limitations and restrictions on individuals’ activities and participation in their society and it was designed to assess the extent of disability associated with a psychiatric condition. It is composed of is 36 items to represent the ICF’s six activity and participation domains: Cognition-understanding and communicating (6 items), Mobility – moving and getting around (5 items), Self-care – hygiene, dressing, eating, and being alone (4 items), Getting along – interacting with other people (5 items), Life activities – domestic responsibilities, leisure, work, and school (8 items), Participation – joining in community activities (8 items). Each item is rated on a 5-point scale from 1 (None) to 5 (Extreme or cannot do) and the patient is asked to indicate how much difficulty did he have in the different areas. The total score is converted into a standard score.

Psychometric properties: The Italian adaptation of WHODAS 2.0 (Federici et al., 2009) has high reliability with Cronbach's alpha ranging from. .69 and .91 in a disable sample and from .48 and .90 in normal participants. The low reliability value obtained in the normal participants' group (Self-care subscale) is probably due to the strong floor effect. WHODAS 2.0 have shown a stable factor structure: for the normal participants group, the total variance explained by six factors is 55.80%; for the disabled participants group, the total variance explained by six factors is 62.76%. We measured the divergent validity in the present sample by correlating WHODAS and WHOQoL scores, reporting a fair high and negative correlation as expected (r = -.64).

Administration time: 20 minutes.

The *UKU Side Effect rating scale* (Lingjaerde et al., 1987)

The *UKU Side Effect rating scale* (Lingjaerde et al., 1987) is a measure of the side effects severity. UKU is a semi-structured interview composed of 48 items scale including three domains (psychic, neurological and autonomic side effect). The examiner evaluates the presence of side effects on a 4-point scale (from 0 (No side effects) to 3 (Side effects that interfere markedly with the patient's performance). A total score is a measure of the severity of side effects.

Administration time: 30 minutes.

Psychometric properties: The UKU is an inclusive instrument with high reported reliability, validity and internal consistency (ICC = .49–.92) (Lingjaerde et al., 1987). We could not test the reliability or concurrent validity for UKU test in our sample therefore we referred only to the above index reported in previous literature.

Tower of London-Drexel University (ToL-DX) (Culbertson and Zillmer, 2001)

The Tower of London-Drexel University (ToL-DX) (Culbertson and Zillmer, 2005) assesses strategic decision-making and planning abilities. The ToL-DX is the most recently developed and marketed version of the ToL (Shallice, 1982). By using a real wooden model with three rods on which three colored balls are placed, the examiner presents ten model of arrangements that the patient is required to copy following specific rules. From the scores available, we selected the four scores which best characterize planning abilities and problem-solving having two scores for planning strategies and two for problem solving: the total number of moves (considered a measure of planning difficulties), the number of correct solutions (considered a measure of efficient problem-solving), time violations (considered a measure of planning inefficiency), and rule violations (considered a measure of inefficient problem-solving). Raw scores are converted into standardized scores and into equivalent scores based on centiles calculated on healthy population (Kaller et al. 2011). Raw scores are converted into standardized scores and into equivalent scores based on centiles calculated on healthy population (Spinnler and Tognoni, 1987).

Administration time: 20 minutes.

Psychometric properties: The Tower of London test also demonstrated good test-retest reliability coefficients, ranging from a low r = .45 to a high r = .81 (Cubertson and Zimmer 2005). We measured the reliability in the present sample with Chronbach's Alpha applied to all the ToL scores and we obtained a quite good index (Chronbach's Alpha = .66).

Modified Wisconsin Card Sorting Test (MCST) (Caffarra et al., 2004)

The Modified Wisconsin Card Sorting Test (MCST) (Caffarra et al., 2004) was used to analyze the tendency towards perseveration and shifting ability. MCST is a shortened version of the Wisconsin Card

Sorting Test (Grant and Berg, 1948) introduced to simplify the task for subjects to which is administered and reduce their frustration and non-compliance (Obonsawin et al. 1999). This test is used as a measure of executive functioning, specifically perseveration and abstract reasoning (Caffarra et al. 2004). It consists of two sets of 24 response cards and four-stimulus card (Obonsawin et al. 1999). The subject is asked to match the response card to specific stimulus card according to specific criteria, including shape, colour and number. The first characteristic chosen by the patient is accepted as correct and, only when the patient achieves six consecutive correct responses, the examiner declares the change of the rule. The test continues until all categories are chosen; then, each category is repeated in the original order. Generally, scores on the number of categories obtained, number of non-perseverative errors, number of perseverative errors and percentage of perseverative errors are available (Obonsawin et al. 1999). However, the Italian normative data are availed only for the number of categories achieved and perseverative errors that are the variables of interest in this study. Raw scores are converted into equivalent scores based on centiles calculated on a healthy population. Generally, a high presence of perseverative errors has a great clinical significance as associated with severe cognitive dysfunctions (Chao et al. 2013).
Administration time: 20 minutes.
Psychometric properties: MCST showed a good test-retest reliability with coefficient r = .67 (Tate et al, 1998), and the split-half reliability estimates of a sample of clinical patients fell into the desirable range (r ≥ .90) (Koop et al., 2019). We measured the reliability in the present sample with Chronbach's Alpha applied to the MCST scores and ee obtained a high index (Chronbach's Alpha = .72).

Attentional Matrices (AM) (Della Sala et al.,1992; **Spinnler and Tognoni, 1987)**

The *Attentional Matrices* (AM) (Della Sala et al.,1992; Spinnler and Tognoni, 1987) test was applied to evaluate selective visual attention. AM is a visual scanning task evaluating selective visual attention. AM is composed of three matrices including 130 numbers from 0 to 9, located randomly on 13 rows and 10 columns (Abbate et al. 2007). The subject is asked to find, within 45 seconds, one or more target numbers. In the first matrix the patient has to recognize one target number; in the second matrix, two target numbers; in the third matrix, three target numbers. Execution time and the number of correct responses are recorded (maximum score of 60) (D'Antiga et al. 2014). Raw scores are converted into equivalent scores based on centiles calculated on healthy population. (Giusti and Murdaca, 2008).

Administration time: 10 minutes.

Psychometric properties: In an Italian sample AM test have shown moderate reliability with a coefficient equal to r = .53 (Spinnler and Tognoni, 1987). We did not have the possibility to test the reliability or concurrent validity for AM test in our sample therefore we referred only to the above index reported in previous literature. We measured the concurrent validity in the present sample by correlating AM and STROOP error scores. We found a negative correlation as expected (r = -.35).

Stroop Colour Word Interference Test (STROOP) (Caffarra et al., 2002)

The *Stroop Colour Word Interference Test* (STROOP) (Caffarra et al., 2002) was used as an index of selective attention, inhibitory control, and processing speed. The version used in our study (Venturini et al. 1983) is composed of three subtests. In the first subtest, the subject is asked to read a list of colours. The second task requires to pronounce the name of these colours. In the third subtest, the subject is asked

to read colored words indicating a specific colour (e.g. the word ―red‖ is written in yellow. Response time of correct response and the errors are recorded. Raw scores are converted into equivalent scores based on centiles calculated on a healthy population.

Administration time: 5-10 minutes.

Psychometric properties: STROOP shown good test-retest reliability (r=.73; p<.001) and high internal consistency (ranging from Cronbach's Alpha=.87 to Cronbach's Alpha=.88) (Siegrist, 1995). We measured the reliability in the present sample with Chronbach's Alpha applied to the STROOP scores. We obtained a low index of reliability (Chronbach's Alpha = .42).

Raven's Coloured Progressive Matrices (CPM-47) Caltagirone et al., 1995)

The Italian standardized version of *Raven's Coloured Progressive Matrices* (CPM-47) (Caltagirone et al., 1995) was used to evaluate fluid intelligence. CPM is a measure assessing intellectual abilities that do not depend on verbal skills, such as visuospatial components and the ability to analyze abstract images according to similarity, dissimilarity, numerical progression and size. It includes three subtests (A, AB, B) of increasing difficulty, each composed of twelve items. In each item, the subject is required to complete a figure by choosing among a set of pieces the one that is logically linked to the whole (Raven, 1984). The sum of correct response is converted into an equivalent score based on centiles calculated on ahealthy population. Low total scores to indicate deficits in observational skills and analogical reasoning.

Administration time: 15-30 minutes.

Psychometric properties: The CPM demonstrated good inter-item consistency (ranging from .76 to .88) and split-half reliability (.81 to .90) (Cotton et al., 2005). We measured the concurrent validity in the present sample by correlating Ravens's CMP and MMSE scores, obtaining a fair good positive relationship (r = .50).

Judgment and Verbal Abstract Tasks test (Test dei Giudizi Verbali e dei Compiti Astratti - GCA, Spinnler and Tognoni, 1987).

The *Judgment and Verbal Abstract Tasks test* (Test dei Giudizi Verbali e dei Compiti Astratti - GCA, Spinnler and Tognoni, 1987) measure mental flexibility and verbal intelligence indicating the quality of reasoning, language and conceptualization skills. It is composed of four subtests concerning the ability to identify: 1) differences in five couples of words; 2) concrete and abstract elements in five proverbs; 3) absurdities in five brief stories; 4) categories for five sets of words. Each item can be rated with a score from 0 (incorrect response) to 3 (correct response). The sum of correct response is converted into equivalent score based on centiles calculated on healthy population (Giusti and Murdaca, 2008). The score 0 indicates a score under the tolerance limit of 5%, thus a pathologic score; the score 4 indicates a performance which is equal or superior to the average; 1 (under the average) 2 (average), 3 (high average) are intermediate scores (Spinnler and Tognoni, 1987).

Administration time: 10 to 15 minutes.

Psychometric properties: moderate reliability of the GCA test has been shown in an Italian sample (Chronbach's Alpha = .43; Spinnler and Tognoni, 1987). We measured the concurrent validity in the present sample by comparing the GCA score to the MMSE score, the correlation showed fair good relationship (r = .53).

Digit Span (SPAN) (Monaco et al., 2013**)**

The Digit Span (SPAN) (Monaco et al, 2013) was used to assess short-term memory (Forward task) and working memory abilities (Backward task). In the forward task, the patient is required to reproduce a sequences of items (digits) of increasing length that are verbally presented by the examiner. The forward task evaluates the functioning of verbal short-term memory. The backward task evaluates the working memory abilities (the patient has to verbally reproduce in the reverse order the sequence of digit presented by the examiner). The sum of total correct item is converted into equivalent score according to centiles calculated on an Italian healthy population. A high equivalent score represents better performance.

Administration time: 10 minutes.

Psychometric properties: Digit span test have shown in an Italian sample a good subscale score test-retest reliability equal to r = .93 for the Working-memory composite score, r = .82 for the forward score and r = .80 for the backward score (Orsini and Pezzuti, 2013). We measured the concurrent validity in the present sample by comparing the SPAN scores to the MMSE score, the correlation showed low correlation (r = .42 and r = .43 for the backward and forward task respectively).

Mini Mental State Examination (MMS-E) (Measso et al., 1993)

The Mini Mental State Examination (MMS-E) is a screening test for the assessment of cognitive impairment (Folstein et al. 1975). The task is composed of 11 items that evaluate the orientation (items 1-2), the short-term memory (item 3), the attention and ability mental calculation (item 4), the long-term memory (item 5) and the language (items 6-11). The total number of correct responses is converted into a standardized score according to normative data (Ruggeri, 1993).

Administration time: 5 -10 minutes.

Psychometric properties: The MMSE test is often used to evaluate the presence of cognitive impairment and to identify the subject with mental disability. For this reason, an Italian research team studied the sensitivity, specificity and AUC of the test (Pirani et al., 2010). MMSE has shown high sensitivity (93%) to recognize subjects with mental deterioration and high specificity (91%) to recognize healthy subjects. Good is the AUC parameter estimated with ROC curves (AUC = .96). High internal consistency (Cronbach's Alpha= .90 to .92) was found and a good test-retest reliability ranging between .80 and .95 (Tombaugh and Mclntyre, 1992; Marioni et al., 2011). We measured the test retest reliability in the present sample by correlating MMSE at admission and MMSE at discharge, reporting fair good correlation (r = .59).

Clock Drawing Test (CDT) (Caffarra et al., 2011**).**

The Clock Drawing Test (CDT) is a neuropsychological instrument for the evaluation of a wide range of cognitive functions, including selective and sustained attention, auditory comprehension, verbal working memory, numerical knowledge, visual memory and reconstruction, visuospatial abilities, on-demand motor execution (praxis) and executive function. The patient is asked to follow a two-step instruction: "First, draw a clock with all the numbers on it. Second, put hands on the clock to make it read 2:45." The scoring represents the ability in the representation of a clock (from 10 to 1). The score is converted into normative score according to normative data. A low score represents more severe general cognitive impairment.
administration time: 5 minutes.

Psychometric properties: CDT has robust psychometric properties: high mean sensitivity (85%) and specificity (85%), high levels of inter-rater (ranging from .75 to .98) and test-retest reliability (.63 to .94; Shulman, 2000). We measured the concurrent validity in the present sample by correlating CDT and MMSE scores, reporting low correlation (r = .48).

Cognitive performance: differences among diagnoses

The Univariate Analysis of Variance showed overlapping performances across diagnoses concerning fluid intelligence ($F_{3,383}$ = 1.20; *p* = .31; *partial* η^2 =.02), ToL total number of moves ($F_{3,383}$ = 2.15; *p* = .09; *partial* η^2 = .017), and time at Stroop test ($F_{3,383}$ = 1.72; *p* = .16; *partial* η^2 =.013). However, diagnoses differed for selective attention at AM ($F_{3,383}$ = 8.33; *p* < .001; *partial* η^2 =.117), categorization ($F_{3,383}$ = 8.06; *p* < .001; *partial* η^2 =.060) and perseveration errors at WCST ($F_{3,383}$ = 5.08; *p* = .02; *partial* η^2 =.038), total number of correct moves ($F_{3,383}$ = 4.30; *p* = .005; *partial* η^2 =.03), rules violation ($F_{3,383}$ = 3.01; *p* = .030; *partial* η^2 =.021), and time violation at TOL ($F_{3,383}$ = 3.67; *p* = .012; *partial* η^2 =.028) and error score at Stroop Test ($F_{3,383}$ = 2.88; *p* = .04; *partial* η^2 =.022). SZ and BD patients showed generalized lower performances as compared to DD and PD. In details, post hoc tests with Bonferroni correction showed that SZ patients had worse performances than DD in perseverative errors (*p* < .001) and categorization (*p* < .001) at MCST, selective attention (*p* < .001) and planning accuracy (*p* = .01). In comparison with PD, SZ patients exhibited lower performances in categorization index (*p* < .001), rule violation at ToL (p < .030) and selective attention (*p* < .001). BD patients performed worse than DD in planning accuracy (*p* = .035) and selective attention (*p* = .042), and worse than PD at Time violation at ToL (*p* = .013). The other post hoc comparisons were not significant. Differences among diagnoses on all cognitive tasks are represented in Figure S1.

Figure S1 Differences in cognitive performances among diagnoses.

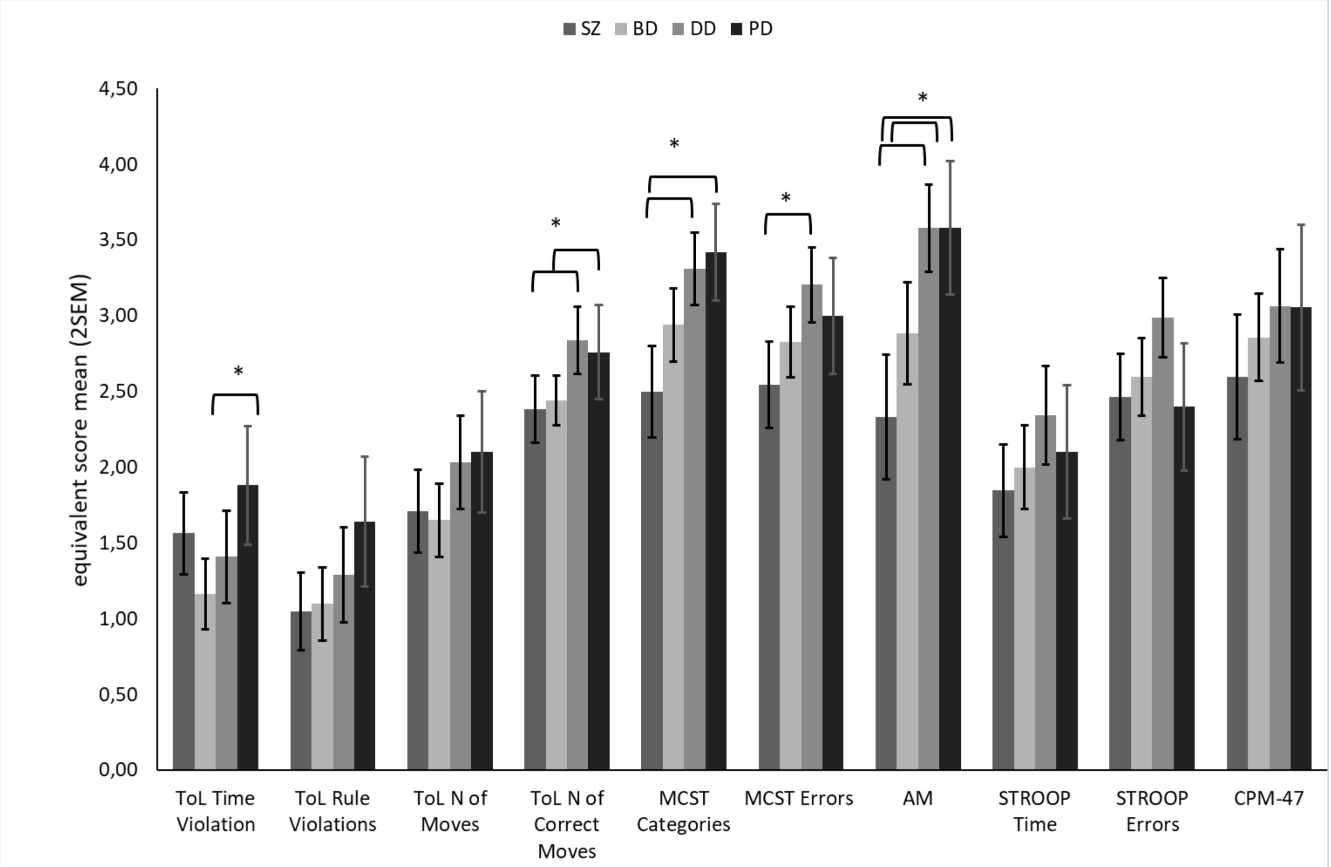

Note: ToL, Tower of London-Drexel University test; MCST, Modified Wisconsin Card Sorting Test; AM, Attention Matrix test; Stroop, Stroop Colour Word Interference Test; CPM-47, Raven's Colored Progressive Matrix.

Table S1 Evidences of cognitive heterogeneity from previous cluster analysis applied to cross-diagnostic samples.

Study	Sample	Assessment	Cluster Analysis	Clustering solution
Goldstein and Shelly (1987)	• Schizophrenia (N=47)<br>• Chronic Alcoholism (N=42)<br>• Psychiatric Disorders (N=36)	Halstead-Reitan battery; Luria-Nebraska battery; Wechsler Adult Intelligence Scale.	- Hierarchical clustering using Ward's method	Four-cluster solution:<br>I. Severe and pervasive impairment;<br>II. Moderate impairment;<br>III. Mild impairment;<br>IV. Normal Performance Levels.
Hermens et al. (2011)	• Anxiety Disorder (N=10)<br>• Depressive Disorder (N=49)<br>• Bipolar Disorder (N=19)<br>• Psychotic Disorder (N=31)<br>All with depressive symptoms.	Semi-structured interviews; Brief Psychiatric Rating Scale; Social and occupational functioning assessment scale; Kessler-10 and Depression anxiety and stress scales; Wechsler Test of Adult Reading; Trail-Making Test - Part B; Wechsler memory scale, third edition; Rey-Osterrieth Complex Figure Test;Rey Auditory Verbal Learning Test; letters subtest of the Controlled Oral Word Association Test; Cambridge Automated Neuropsychological Testing Battery; Total adjusted errors score from the Intra Dimensional/Extra-Dimensional task; Rapid Visual Information Processing task; Total adjusted errors score from the Paired Associate Learning task; Span length score from the Spatial Span task.	- Hierarchical clustering using Ward's method	Three-cluster solution:<br>I. Poor visual and verbal memory;<br>II. Severely impaired, especially in mental flexibility, verbal learning and memory;<br>III. Poor mental flexibility with other performances preserved.
Lewandowski et. al (2014)	• Schizophrenia (N=41)<br>• Psychotic Bipolar Disorder (N=73)<br>• Schizoaffective Disorder (N=53)	Visuospatial memory test; Stroop color and word test; Hopkins verbal learning test-Revised; Total recall and delayed recall measures; Category fluency; Positive and negative syndrome scale; Young mania rating scale; Montgomery-Asberg depression rating scale; Multnomah community	- Hierarchical clustering using Ward's method<br>- Non-hierarchical clustering using K-means method	Four-cluster solution:<br>I. Neuropsychologically normal;<br>II. Severe visuospatial impairment, moderate verbal impairment;<br>III. Poor verbal and executive functioning;<br>IV. Severe impairment,

Lee et al. (2015)	• Major Depression (N=71)<br>• Bipolar Disorder (N=61)<br>• Schizophrenia-Spectrum (N=35)<br>• Healthy (N=63)	Wechsler Test of Adult Reading; Wide Range Achievement Test; Trail Making Test—Part A; Logical Memory I and Logical Memory II; Percent Retention Rapid Visual Processing Hits A'; Paired Associate Learning adjusted errors; Intra-/Extradimensional shift test; Controlled Oral Word Association Test; World Health Organization Disability Assessment Scale version 2.0 ; World Health Organization Quality of Life.	- Hierarchical clustering using Ward's method<br>- Non-hierarchical clustering using K-means method	Three-cluster solution:<br>I. Reductions in psychomotor speed;<br>II. Improvement in sustained attention;<br>III. Improvement in verbal memory.
Reser et al. (2015)	• Schizophrenia (N=51)<br>• Schizophreniform (N=9)<br>• Schizoaffective (N=16)<br>• Delusional Disorder (N=6)<br>• Brief Psychotic Disorder (N=1)<br>• Psychotic Disorder (N=15)	WAIS-III digit Span and Letter-Number Sequencing subscales; Delayed verbal memory Rey Auditory Verbal Learning Test, A7; Delayed non- verbal memory Rey-Osterrieth Complex Figure Tests, Delayed Recall; RCF: Recognition Trial; Attention and processing speed Trail Making Test A and B; Letter Cancellation task; Symbol Digit Modalities Test; Language functions Category Fluency Test: Animal; Control Oral Word Association Test: Letters F-A-S subscale; Visuo-spatial ability RCF: Copy condition; Executive functioning RCF; Organisational Strategy Score.	- Hierarchical clustering using Ward's method<br>- Non-hierarchical clustering using K-means method	Four-cluster solution:<br>I. High attention, working memory, visual recognition;<br>II. Low in attention and working memory but strong in visual recognition;<br>III. Intact in almost all domains;<br>IV. Lowest in almost all domains.
Van Rheenen et al. (2017)	• Schizophrenia (N=564)<br>• Bipolar Disorder (N=402)<br>• Healthy (N=575)	MATRICS Consensus Cognitive Battery (MCCB; Kern et al. 2008) included measures of: speed of processing; attention/vigilance; working memory; visual learning; reasoning and problem solving; and social cognition; verbal learning.	- Hierarchical clustering using Ward's method	Three-cluster solution:<br>I. Severely impaired;<br>II. Mild-moderately impaired;<br>III. Relatively intact.

Cotrena et al. (2017)	• Bipolar Disorder I or II (N=54)<br>• Major Depressive Disorder (N=33)	Stroop Color Word Test; Hayling Sentence Completion Test; and the Trail Making Test; Sentence-Word Span; Backwards Digit span; Montreal Communication Assessment Battery; Divided Attention Test; Cancellation Task.	- Hierarchical clustering using Ward's method	Three-cluster solution:<br>I. Above-average on all domains;<br>II. Worse than cluster 1 plus motor and verbal impairment;<br>III. Severe impairment in verbal inhibition and cognitive flexibility.
Lee et al. (2017)	• Bipolar Disorder I or II (N=68)<br>• Schizophrenia (N=39)<br>• Healthy (N36)	A range of electrophysiological and performance-based assessments measured perception, nonsocial cognition and social cognition on auditory and visual modalities. Behavioral measures assessed early visual perception, nonsocial cognition and social cognition.	- Non-hierarchical clustering using K-means method	Two-cluster solution:<br>I. Relatively preserved social cognition;<br>II. Impaired social cognition.
Crouse et al., 2018	• Schizophrenia Spectrum (N=90)<br>• Affective Spectrum (N=44)<br>• Healthy (N=50)	Wechsler Test of Adult Reading; Trail-Making Test Part-A&Part-B; Rey Auditory Verbal Learn- ing Test; Controlled Oral Word Association Test; Rapid Visual Processing A', Intra-Extra Dimensional Set Shift, Spatial Span task, Paired Associates Learning tests-	- Hierarchical clustering using Ward's method	Three-cluster solution:<br>I. Superior socio-occupational functioning and highest premorbid IQ;<br>II. Lower premorbid IQ;<br>III. Lower premorbid IQ, cognitive impairment.

Lewandowski, 2018	• Mood Disorders with Psychosis (N=64)<br>• Schizoaffective Disorder (N= 28)<br>• Schizophrenia (N=21)	Trail Making Test A; Brief Assessment of Cognition in Schizophrenia: Symbol Coding; Category Fluency; Continuous Performance Test: Identical Pairs; Wechsler Memory Scale Spatial Span; Letter Number Span; Brief Visuospatial Memory Test; Hopkins Verbal Learning Test); Neuropsychological Assessment Battery: Mazes; Mayer-Salovey-Caruso Emotional Intelligence Test: Managing Emotions.	- Hierarchical clustering using Ward's method	Four-cluster solution:<br>I. Neuropsychologically normal;<br>II. Mild impairment in processing speed, attention, verbal, visual and executive functions;<br>III. Mild executive and social impairment; moderate processing speed and attention impairment;<br>IV. Globally impaired.

Figure S2. AIC BIC and Entropy change from Latent Class analysis and Two-Step cluster analysis for solutions ranging from 1 to 4 clusters in all diagnoses.

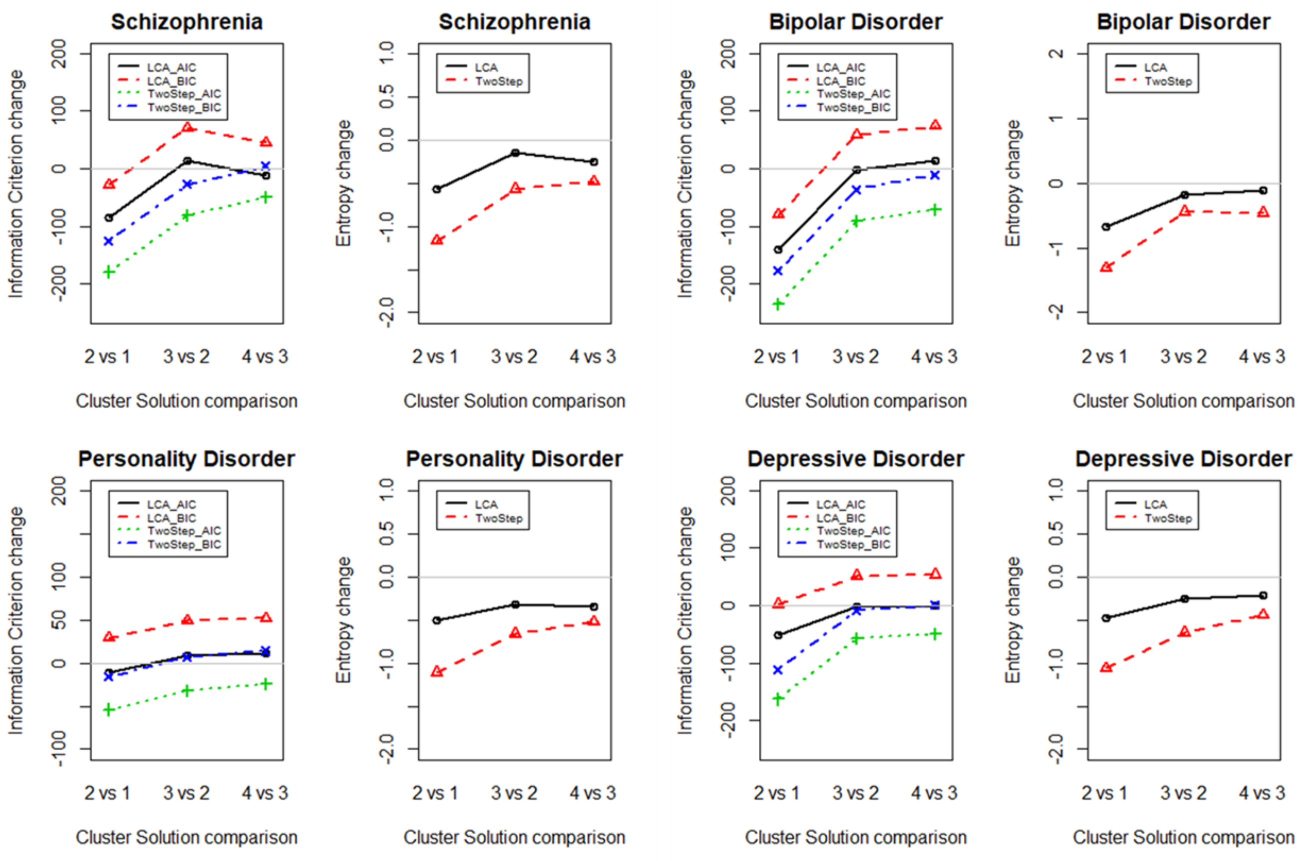

Note: The panels show the change in Information Criterion (left) or Entropy (right) between two close clusters solutions (e.g., 2vs1 shows 2-cluster solution minus the 1-cluster solution). LCA, Latent Class cluster analysis; TwoStep, Two-Step cluster analysis; BIC, Bayesian Information Criterion; AIC, Akaike Information Criterion.

Table S2. Bayesian Information Criterion (BIC), Akaike Information Criterion (AIC) and Entropy for the cross- diagnostic sample and within each diagnosis as emerged from Two-Step and Latent Class cluster analysis.

		TWO-STEP			LATENT CLASS		
	**N Clusters**	**BIC**	**AIC**	**ENTROPY**	**BIC**	**AIC**	**ENTROPY**
Cross-DIAGNOSTIC	1	7572	7493	9.6	7572	7493	9.6
	2	7041	6877	8.5	7291	7129	9.1
	3	6875	6626	8.1	7326	7081	9.0
	4	6749	6379	7.7	7366	7037	8.9
Schizophrenia	1	2227	2173	9.7	2227	2173	9.7
	2	2102	1994	8.5	2199	2089	9.1
	3	2075	1913	8.0	2251	2084	8.9
	4	2080	1864	7.5	2342	2118	8.8
Bipolar Disorder	1	2640	2582	9.5	2640	2582	9.4
	2	2463	2347	8.2	2560	2441	8.8
	3	2427	2256	7.7	2629	2449	8.6
	4	2417	2185	7.3	2677	2436	8.4
Depressive Disorder	1	1786	1736	9.1	1786	1736	9.1
	2	1675	1573	8.1	1788	1685	8.6
	3	1667	1515	7.4	1837	1680	8.3
	4	1668	1465	7.0	1905	1695	8.2
Personality Disorder	1	1013	975	9.4	1013	975	9.3
	2	997	921	8.2	1041	963	8.8
	3	1004	889	7.6	1096	977	8.5
	4	1018	865	7.1	1156	997	8.2

Table S3. Description of the two clusters within the diagnosis of Schizophrenia and Bipolar Disorder according to the number and percentage of cases scoring below, within, and above the normative scores for each cognitive test.

		Schizophrenia						***Bipolar Disorder***					
		**Cluster 1**			**Cluster 2**			***Cluster 1***			**Cluster 2**		
		below	***within***	***above***	***below***	***within***	***above***	***below***	***within***	***above***	***below***	***within***	***above***
TWO-STEP	*CPM47*	19	14	2	8	31	36	4	18	46	22	34	10
	AM	23	12	0	17	33	25	6	21	41	32	22	12
	STROOP Time	20	12	3	31	25	19	13	30	25	40	13	13
	STROOP Errors	19	15	1	9	27	39	4	29	35	28	17	21
	ToL N of Correct Moves	11	22	2	15	49	11	4	52	12	19	45	2
	ToL Total N of Moves	29	5	1	22	45	8	13	46	9	52	13	1
	ToL Rule Violations	34	1	0	37	36	2	28	37	3	56	8	2
	ToL Time Violations	32	3	0	22	52	1	23	44	1	62	4	0
	MCST Categories	19	12	4	15	17	43	4	12	52	22	23	21
	MCST Errors	17	10	8	12	31	32	4	26	38	19	31	16
LATENT CLASS	*CPM47*	28	2	14	10	25	31	46	1	14	10	25	38
	AM	18	5	21	7	35	24	38	3	20	15	35	23
	STROOP Time	12	13	19	10	38	18	23	11	27	15	42	16
	STROOP Errors	21	4	19	19	24	23	32	3	26	24	29	20
	ToL N of Correct Moves	10	6	28	3	20	43	11	5	45	3	18	52
	ToL Total N of Moves	8	10	26	1	41	24	8	12	41	2	52	18
	ToL Rule Violations	2	13	29	0	58	8	3	22	36	2	62	9

	ToL Time Violations	1	10	33	44	22	0	1	24	36	61	12	0
	MCST Categories	35	1	8	12	33	21	52	1	8	21	25	27
	MCST Errors	25	3	16	15	26	25	38	1	22	16	22	35

Table S4. Description of the two clusters within the diagnosis of Depressive Disorder and Personality Disorder according to the number and percentage of cases scoring below, within, and above the normative scores for each cognitive test.

		Depressive Disorder						*Personality Disorder*					
		**Cluster 1**			**Cluster 2**			***Cluster 1***			**Cluster 2**		
		below	***within***	***above***	***below***	***within***	***above***	***below***	***within***	***above***	***below***	***within***	***above***
TWO-STEP	*CPM47*	1	6	27	10	25	24	4	10	18	2	13	3
	AM	1	8	25	15	15	29	9	11	12	6	6	6
	STROOP Time	8	14	12	20	19	20	8	14	10	11	3	4
	STROOP Errors	0	12	22	11	22	26	8	11	13	5	9	4
	ToL N of Correct Moves	2	20	12	5	49	5	0	24	8	6	10	2
	ToL Total N of Moves	4	20	10	27	32	0	5	22	5	11	7	0
	ToL Rule Violations	1	27	6	52	7	0	13	18	1	12	5	1
	ToL Time Violations	11	19	4	41	16	2	7	24	1	12	6	0
	MCST Categories	2	1	31	7	22	30	0	1	31	6	7	5
	MCST Errors	2	3	29	7	27	25	1	8	23	7	7	4
LATENT CLASS	*CPM47*	45	2	20	6	9	11	18	2	11	3	4	12
	AM	43	5	19	11	11	4	14	5	12	4	10	5
	STROOP Time	26	12	29	6	16	4	10	6	15	4	3	12
	STROOP Errors	36	4	27	12	7	7	12	7	12	5	6	8
	ToL N of Correct Moves	17	4	46	3	23	0	10	0	21	6	13	0

ToL Total N of Moves	10	17	40	0	14	12	4	6	21	1	10	8
ToL Rule Violations	6	29	32	24	2	0	2	12	17	13	6	0
ToL Time Violations	5	30	32	1	22	3	1	8	22	0	11	8
MCST Categories	53	3	11	8	6	12	29	1	1	7	5	7
MCST Errors	47	3	17	7	6	13	23	2	6	4	6	9

Table S5. Results of Two-Step cluster analysis applied to continuous standardized scores: Schwarz's Bayesian Information Criterion (BIC), BIC Change, Akaike's Information Criterion (AIC), AIC Chang are reported for different cluster solution.

Cluster solution	BIC	BIC Change	AIC	AIC Change
1	1459.124		1393.562	
2	1268.824	-190.300	1137.699	-255.863
3	1282.474	13.650	1085.787	-51.913
4	1301.360	18.886	1039.111	-46.676
5	1348.354	46.994	1020.543	-18.568
6	1419.654	71.300	1026.281	5.738
7	1491.751	72.097	1032.815	6.535
8	1566.147	74.396	1041.649	8.834
9	1641.744	75.597	1051.684	10.035
10	1718.417	76.672	1062.794	11.110
11	1797.873	79.456	1076.688	13.894
12	1878.796	80.923	1092.048	15.361
13	1962.320	83.525	1110.011	17.962
14	2045.947	83.627	1128.075	18.064
15	2134.284	88.337	1150.850	22.775

REFERENCES

Abbate, C., Luttazzi, C., and Vergani, C. (2007). Test delle matrici: velocità e accuratezza della ricerca visiva nel corso dell'invecchiamento. *G Gerontologia*, 55, 11–20.

Caltagirone, C., Gainotti, G., Carlesimo, G. A., & Parnetti, L. (1995). il Gruppo per la standardizzazione della Batteria per il Deterioramento Mentale,". Batteria per la valutazione del Deterioramento Mentale (parte I): descrizione di uno strumento di diagnosi neuropsicologica, 461-470.

Caffarra, P., Gardini, S., Zonato, F., Concari, L., Dieci, F., Copelli, S., ... & Venneri, A. (2011). Italian norms for the Freedman version of the Clock Drawing Test. *Journal of Clinical and Experimental Neuropsychology, 33*, 982-988.

Caffarra, P., Vezzadini, G., Dieci, F., Zonato, F., & Venneri, A. (2002). Una versione abbreviata del test di Stroop: Dati normativi nella popolazione Italiana. *Nuova Rivista di Neurologia, 12*(4), 111-115.Chao, J.-K., Hao, L.-J., Chao, I.-C., Shi, M.-D., and Chao, I.-H.C. 2013. Utility of nelson's modified card sorting test in patients with alzheimer's disease or vascular dementia. *Open Journal of Preventive Medicine*, 03, 172–177.

Cotton, S. & Kiely, P., Crewther, D., Thomson, B., Laycock, R., and Crewther, S. (2005). A normative and reliability study for the Raven's Coloured Progressive Matrices for primary school aged children from Victoria, Australia. *Personality and Individual Differences*. 39. 647-659.

Culbertson, W. C., and Zilmer E. A. (1998). The Construct Validity of The Tower of London DX As a Measure of The Executive Functioning of ADHD Children. *Assessment* 5, 215–26.

Culbertson, W. C., & Zillmer, E. A. (2001). The Tower of London DX (TOL DX) manual. North Tonawanda, NY: Multi-Health Systems.

D'Antiga, L., Dacchille, P., Boniver, C., Poledri, S., Schiff, S., Zancan, L., and Amodio, P. (2014). Clues for minimal hepatic encephalopathy in children with noncirrhotic portal hypertension. *J. Pediatr. Gastroenterol. Nutr.*, 59, 689–694.

Della Sala, S., Laiacona, M., Spinnler, H., & Ubezio, C. (1992). A cancellation test: its reliability in assessing attentional deficits in Alzheimer's disease. *Psychological Medicine*, 22, 885-901.

De Girolamo, G., Bellini, M., Bocchia, S., and Ruggeri, M. (1995). "Introduzione." *Epidemiologia e Psichiatria Sociale* 4, 69–71.

Falco, Fabrizio Antonio et al. 2008. "The Neurologist in the Emergency Department. An Italian Nationwide Epidemiological Survey." *Neurological Sciences* 29(2): 67–75.

Federici, S., Meloni, F., Mancini, A., Lauriola, M., and Olivetti Belardinelli, M. (2009). World Health Organisation disability assessment schedule II: Contribution to the Italian validation. *Disability and rehabilitation*, 31(7), 553-564.

Gigantesco, A, Picardi, A, de Girolamo, G. B., and Morosini, P. (2007). Discriminant Ability and Criterion Validity of the HoNOS, *Italian Psychiatric Residential Facilities Psychopathology*, 40,111–115.

Gignac, G. E., Reynolds, M. R., and Kovacs, K. (2019). "Digit Span Subscale Scores May Be Insufficiently Reliable for Clinical Interpretation: Distinguishing Between Stratified Coefficient Alpha and Omega Hierarchical." *Assessment* 26, 1554–63.

Girolamo, G., De et al. (2000). "Quality of Life Assessment: Validation of the Italian Version of the WHOQOL-Brief." *Epidemiologia e Psichiatria Sociale* 9,: 45–55.

Giusti, E., and Murdaca, F. (2008). Psicogerontologia. Interventi piscologici integrati in tarda età Sovera Edizioni.

Grant, D., and Berg, E.A. (1948). A behavioral analysis of degree of impairment and ease of shifting to new responses in a Weigl-type card sorting problem. *Journal of Experimental Psychology* 39, 404–411.

Kaller, C. P., Unterrainer, J. M., and Stahl, C. (2012). "Assessing Planning Ability with the Tower of London Task: Psychometric Properties of a Structurally Balanced Problem Set." *Psychological Assessment* 24, 46–53.

Lingjærde, O., Ahlfors, U.G., Bech, P., Dencker, S. and Elgen, K. (1987), The UKU side effect rating scale: A new comprehensive rating scale for psychotropic drugs and a cross-sectional study of side effects in neuroleptic-treated patients. Acta Psychiatrica Scandinavica, 76: 1-100. Marioni, R. E., Chatfield, M., Brayne, C., and Matthews, F. E. (2011). The Reliability of Assigning Individuals to Cognitive States Using the Mini Mental-State Examination: A Population-Based Prospective Cohort Study. *BMC Medical Research Methodology*, 11.

Measso, G., Cavarzeran, F., Zappala, G., Lebowitz, B. D., Crook, T. H., Pirozzolo, F. J., et al., (1993). The mini-mental state examination: Normative study of an Italian random sample. *Developmental neuropsychology*, 9(2), 77-85.

Monaco, M., Costa, A., Caltagirone, C., & Carlesimo, G. A. (2013). Forward and backward span for verbal and visuo-spatial data: standardization and normative data from an Italian adult population. *Neurological Sciences*, 34(5), 749-754.

Morosini P., Casacchia M. (1995). Brief psychiatric rating scale BPRS versione 4.0 ampliata. Manuale di istruzioni. Adattamento italiano.

Morosini, P., Gigantesco A., Mazzarda A., and Gibaldi L. (2003). "HoNOS-Roma: Una Versione Ampliata, Personalizzabile e Che Facilita La Compilazione Ripetuta Nel Tempo Dello Strumento HoNOS." *Epidemiologia e Psichiatria Sociale*, 12, 53–62.

Mortimer, A. M. (2007). Symptom rating scales and outcome in schizophrenia. *British Journal of Psychiatry Supplement*, 50, s7–s14.

Nuovo, S. D. (2006). La valutazione dell’attenzione. Dalla ricerca sperimentale ai contesti applicativi FrancoAngeli.

Orsini, A., and Pezzuti, L. 2013. WAIS Contributo alla taratura italiana (16-69). Firenze: Giunti OS

Obonsawin, M. C., Crawford, J. R., Page, J., Chalmers, P., et al. (1999). Performance on the Modified Card Sorting Test by normal, healthy individuals: Relationship to general intellectual ability and demographic variables. *The British Journal of Clinical Psychology,* 38, 27–41.

Pirani, A., Brodaty, H., Martini, E., Zaccherini, D., Neviani, F., & Neri, M. (2010). The validation of the Italian version of the GPCOG (GPCOG-It): A contribution to cross-national implementation of a screening test for dementia in general practice. *International Psychogeriatrics*, 22, 82-90.

Pruneti, C.A. (1985). Dati Normativi del Test PM 47 Coloured su un campione di bambini italiani Normative data of CPM 47 on a large sample of Italian schoolchildren. *Bollettino Di Psicologia Applicata,* 27–35.

Raven, J. C. (1984). CPM. Coloured Progressive Matrices. Giunti OS, Firenze.

Roncone, R., Ventura, J., Impallomeni, M., Falloon, I.R.H., Morosini, P.L., Chiaravalle, E. and Casacchia, M. (1999), Reliability of an Italian standardized and expanded Brief Psychiatric Rating Scale (BPRS 4.0) in raters with high vs. low clinical experience. *Acta Psychiatrica Scandinavica,* 100, 229-236.

Seigerschmidt, E. Mösch, E., Siemen, M., Förstl, H., and Bickel, H.. (2002). “The Clock Drawing Test and Questionable Dementia: Reliability and Validity.” *International Journal of Geriatric Psychiatry* 17, 1048–54.

Shallice, T. 1982. Specific impairments of planning. Philosophical Transaction 298, 199–209.

Shulman, K. I. (2000). “Clock-Drawing: Is It the Ideal Cognitive Screening Test?” *International Journal of Geriatric Psychiatry* 15(6): 548–61.

Siegrist, M. (1995). “Reliability of the Stroop Test with Single-Stimulus Presentation.” *Perceptual and motor skills* 81, 1295–98.

Spinnler, H., and Tognoni, G. (1987). Italian standardization and classification of Neuropsychological tests. *Italian Journal of Neurological Science*, 8, 1.

Tate, R.L., Perdices M., Maggiotto S. (1998). Stability of the Wisconsin Card Sorting Test and the Determination of Reliability of Change in Scores. *Clinical Neuropsychologist* 12(3): 348–57.

Tombaugh T.N., McIntyre N.J. (1992). The mini-mental state examination: a comprehensive review. J Am Geriatr Soc, 409, 922-935.

Tombaugh T. N., and Mclntyre N. J. (1992). “The Mini-Mental State Examination :” *Progress in Geriatrics* 40(922), 922–35.

Venturini, R., Lombardo Radice, M., and Imperiali, M. G. (1983). Color-word - o test di Stroop. Firenze.

Ware J. E., and Sherbourne C. D. (1992). The MOS 36 Item Short-Form Health Status survey. I. Conceptual framework and item selection. *Medical Care* 30, 473-483.

Wechsler, D. (2008). Wechsler Adult Intelligence Scale–Fourth edition: Technical and interpretive manual. San Antonio, TX: Pearson Assessment.
